# Odour-induced analgesia mediated by hypothalamic orexin neurons in mice

**DOI:** 10.1038/srep37129

**Published:** 2016-11-15

**Authors:** Shogo Tashiro, Ran Yamaguchi, Sodemi Ishikawa, Takeshi Sakurai, Katsuko Kajiya, Yuichi Kanmura, Tomoyuki Kuwaki, Hideki Kashiwadani

**Affiliations:** 1 Department of Physiology, Graduate School of Medical and Dental Sciences, Kagoshima University, 8-35-1 Sakuragaoka, Kagoshima 890-8544, Japan; 2 Department of Anesthesiology, Graduate School of Medical and Dental Sciences, Kagoshima University, 8-35-1 Sakuragaoka, Kagoshima 890-8544, Japan; 3 Department of Biochemical and Nutritional Chemistry, Faculty of Agriculture, Kagoshima University, 1-21-24 Korimoto, Kagoshima 890-0065, Japan; 4 Department of Molecular Neuroscience and Integrative Physiology, Faculty of Medicine, Kanazawa University, 13-1 Takaramachi, Ishikawa 920-8640, Japan

## Abstract

Various folk remedies employ certain odorous compounds with analgesic effects. In fact, linalool, a monoterpene alcohol found in lavender extracts, has been found to attenuate pain responses via subcutaneous, intraperitoneal, intrathecal, and oral administration. However, the analgesic effects of odorous compounds mediated by olfaction have not been thoroughly examined. We performed behavioural pain tests under odourant vapour exposure in mice. Among six odourant molecules examined, linalool significantly increased the pain threshold and attenuated pain behaviours. Olfactory bulb or epithelium lesion removed these effects, indicating that olfactory sensory input triggered the effects. Furthermore, immunohistochemical analysis revealed that linalool activated hypothalamic orexin neurons, one of the key mediators for pain processing. Formalin tests in orexin neuron-ablated and orexin peptide-deficient mice showed orexinergic transmission was essential for linalool odour-induced analgesia. Together, these findings reveal central analgesic circuits triggered by olfactory input in the mammalian brain and support a potential therapeutic approach for treating pain with linalool odour stimulation.

Various natural extracts or their chemical components have long been examined for pain relief. In terms of folk remedies, several odorous compounds extracted from plants are believed to have analgesic effects. For example, linalool, one of the major odorous components of lavender extracts, attenuates pain responses via subcutaneous^1^,^2^,^3^, intraperitoneal^4^,^5^, intrathecal^5^, or oral administration^5^. However, the analgesic effects of odorous compounds mediated by olfaction have been poorly examined.

Odourants inhaled into the nostrils are first detected by olfactory sensory neurons (OSN) in the olfactory epithelium (OE) via odourant receptors^6^. OSNs project their axons to the ipsilateral main olfactory bulb (MOB), the first relay station of the olfactory system. Mitral and tufted cells, the projection neurons in the MOB, receive excitatory synaptic input from OSNs and transmit olfactory sensory information to olfactory cortices^7^. Pyramidal cells in olfactory cortices receive excitatory synaptic input from mitral and tufted cells and directly project their axons to the hypothalamus or indirectly via the amygdala or frontal cortex^8^,^9^. Through these passages, olfactory inputs affect information processing in the hypothalamus and might modulate behaviour, mood, and autonomic functions.

The hypothalamus is an integrative centre for autonomic and other essential functions related to survival, including analgesia. Originating from the discovery that the hypothalamus is involved in stimulation-induced analgesia^10^,^11^, regulatory mechanisms involving the hypothalamus on the descending modulation of pain have been extensively studied^12^. Although various types of peptidergic hypothalamic neurons contribute to analgesic effects^12^, orexin-containing neurons have recently been implicated in analgesia^13^,^14^,^15^,^16^,^17^. Orexin was first identified as a hypothalamic neuropeptide involved in regulating feeding behaviour^18^; however, it is now known to regulate a wide range of fight-or-flight responses^19^. Both orexin-A and orexin-B are derived from a common precursor, prepro-orexin^18^, and both isoforms of orexin peptide contribute to analgesia^13^.

The present study observed that linalool vapour exposure, one of the monoterpene alcohols, induced analgesic effects in mice. However, analgesic effects were not observed in two types of distinct anosmic model mice, indicating that the effects were triggered by olfactory input evoked by linalool vapour. Furthermore, we found that the analgesic effects were mediated by hypothalamic orexin neurons. These findings suggest analgesic circuits triggered by olfactory input might be inherent in the mammalian brain.

## Results

### Linalool vapour exposure attenuates pain behaviours

**Figure 1:**
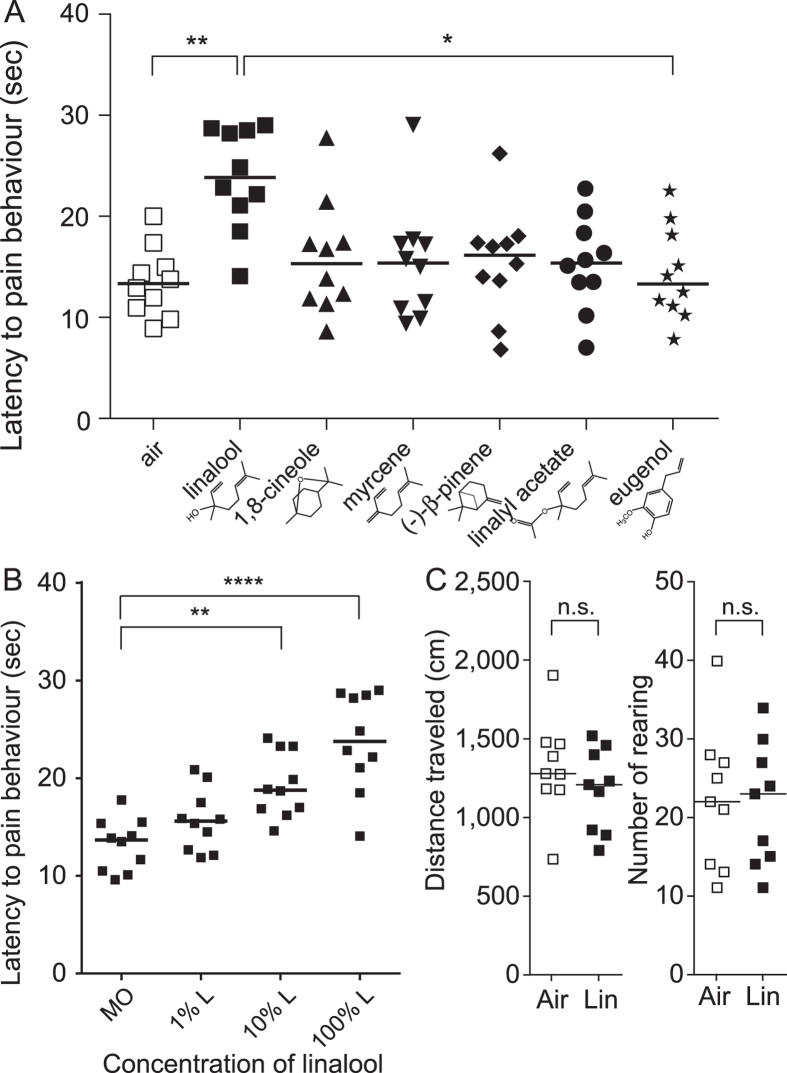
Linalool vapour exposure induces analgesic effects in the hot plate test. (**A**) Increase in pain threshold to a noxious heat stimulus during linalool vapour exposure. Among the odour molecules examined, linalool exposure significantly increased latency to pain responses during the hot plate test; *n* = 10 for each group. (**B**) Concentration-dependent increase of the analgesic effects; *n* = 10 for each concentration. The data set for 100% linalool was the same as the data for linalool group in (**A**) and shown again for comparison. (**C**) No significant changes of spontaneous activity under linalool vapour exposure; *n* = 9 for each group. MO, diluent mineral oil; 1% L, 1% linalool; 10% L, 10% linalool; 100% L, 100% linalool; Air, odourless air-exposed mice; Lin, linalool-exposed mice. Bars indicate the median of each group. *p < 0.05; **p < 0.01; ****p < 0.0001; n.s., not significant.

As an initial screening test of the odorous molecules showing potential analgesic effects, we performed the hot plate test under odour exposure. Latency of hind paw withdrawal was significantly different among the six odour molecules examined (linalool and five structurally related odorous molecules) (*H* = 17.24, *k* = 7, *p* < 0.01). Post hoc Dunn’s multiple comparison test revealed that the latency significantly increased (mean rank difference = 33.45, *p* < 0.01) in linalool vapour-exposed mice compared with odourless air-exposed mice (Fig. 1A). The latency was not significantly altered by the other five odour molecules. These results indicate that linalool vapour exposure increased the pain threshold for a noxious heat stimulus. Next, to examine the concentration-dependency of the linalool vapour-induced analgesia, we assessed the effect at different linalool concentrations. The effect increased with the increase of the linalool concentration, and the effect was significant at 10% (mean rank difference = 16.15, *p* < 0.01) and 100% linalool (mean rank difference = 22.55, p < 0.0001) (Fig. 1B). Furthermore, the spontaneous activity analyses indicated that the linalool vapour exposure did not have significant effects to total distance travelled and number of rearing (Fig. 1C), suggesting that the linalool vapour exposure did not impair the motor function.

**Figure 2:**
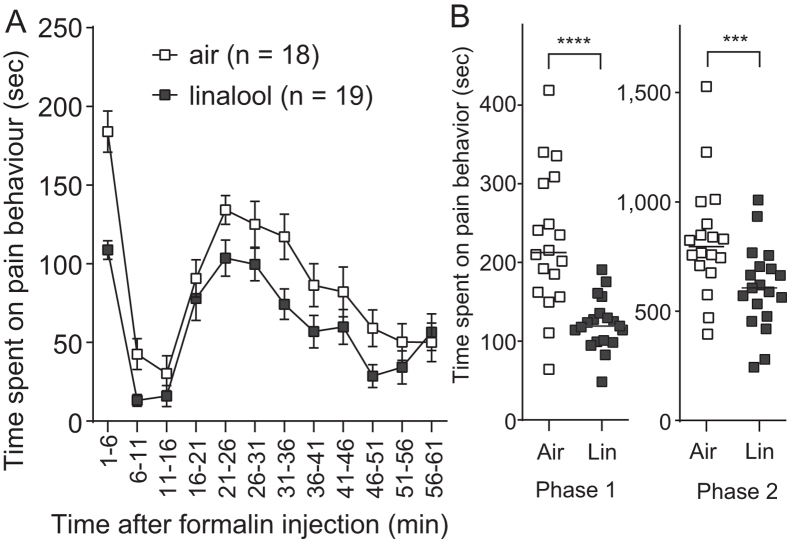
Attenuation of formalin-evoked pain responses during linalool vapour exposure. (**A**) Time course of formalin-evoked pain responses among odourless air-exposed (white squares) and linalool-exposed (black squares) mice. Traces are presented as mean ± SEM. (**B**) A population analysis of linalool-induced analgesia during the formalin test. Linalool significantly suppressed pain responses during both Phases 1 and 2 during the formalin test. Air, odourless air-exposed mice; Lin, linalool-exposed mice; *n* = 18 for odourless air-exposed mice and *n* = 19 for linalool exposed mice. Bars in (**B**) indicate the median of each group. ***p < 0.005; ****p < 0.0001.

We further examined the analgesic effect of linalool vapour on irritant-evoked pain behaviour using a formalin test. Subcutaneous injection of 2% formalin into a hind paw resulted in typical biphasic pain responses^20^ among both linalool vapour-exposed and odourless air-exposed mice (Fig. 2A). However, the time spent in pain behaviour was significantly reduced in linalool vapour-exposed mice during both the first and second phases (1–11 min and 11–61 min after injection, respectively). The duration of pain behaviour significantly decreased during both the first (*U* = 42, *n* = 18 and 19, *p* < 0.0001) and second (*U* = 77, *n* = 18 and 19, *p* < 0.005) phases in linalool vapour-exposed mice compared with odourless air-exposed mice (Fig. 2B). Therefore, linalool vapour exposure attenuated formalin-evoked acute and chronic pain behaviours. From these results, we concluded that linalool vapour exposure induced analgesic effects in mice.

### Olfactory inputs are essential for linalool odour-induced analgesia

**Figure 3:**
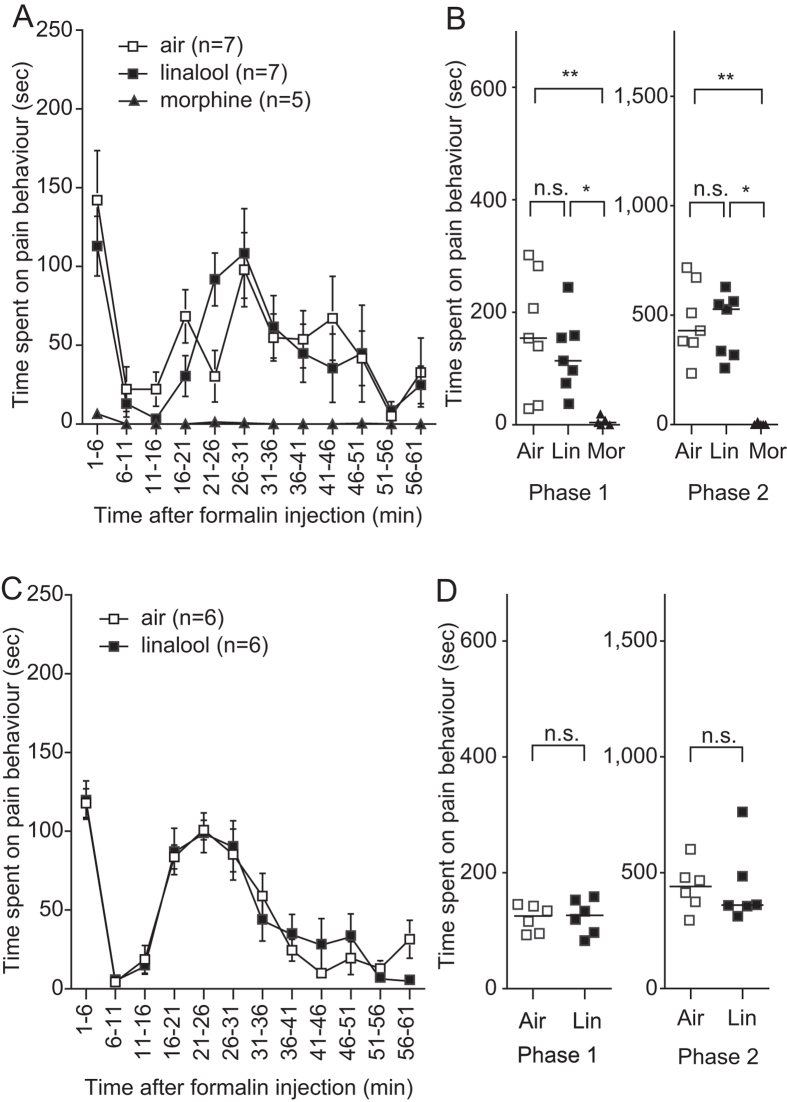
Anosmic model mice do not show linalool-induced analgesia. (**A**) Time course of formalin-evoked pain responses among olfactory bulbectomy (OB) mice. Linalool-induced analgesia is not observed among OB mice. Pain responses are attenuated after subcutaneous morphine injection in OB mice during odourless-air exposure. (**B**) A population analysis of linalool-induced analgesia among OB mice. The time spent on pain behaviour is unchanged in the linalool-exposed group but significantly reduced in the morphine-administered, odourless air-exposed group during both Phases 1 and 2. (**C**) Time course of formalin-evoked pain responses among olfactory epithelium deprived (3-MI) mice. Attenuation of pain responses induced by linalool-exposure is not observed in 3-MI mice. (**D**) A population analysis of linalool-induced analgesia in 3-MI mice. Time spent on pain behaviour in linalool-exposed mice does not show significant differences compared with control mice. Air, odourless air-exposed mice; Lin, linalool-exposed mice; Mor, morphine-administered mice. Traces are presented as mean ± SEM. Bars in plots indicate the median of each group; *n* = 7 for odourless air-exposed OB mice; *n* = 7 for linalool-exposed OB mice; *n* = 5 for morphine-administered OB mice; *n* = 6 for odourless air-exposed 3-MI mice; *n* = 6 for linalool-exposed 3-MI mice.

Next, we examined whether linalool-induced analgesia depended on olfactory processing. We disrupted olfactory information flow through bilateral olfactory bulb suctioning (olfactory bulbectomy) and assessed the effect of linalool vapour exposure using the formalin test. After bulbectomy, the time course of pain behaviours in linalool vapour-exposed mice was not different from that in odourless air-exposed mice (Fig. 3A). Quantitative analyses showed no significant difference between the linalool group and the odourless air group in the time spent exhibiting pain behaviours during both the first and second phases (Fig. 3B). This result suggests that the olfactory input evoked by linalool vapour drove the analgesic system and attenuated pain responses. However, pre-treatment with morphine (10 mg/kg, i.p.), significantly attenuated pain responses in mice that had undergone bulbectomy, indicating that the opioid-mediated analgesic system was intact (Fig. 3A,B).

To confirm the contribution of olfactory input to odour-induced analgesia, we made another anosmic model mouse with non-functional olfactory epithelium (OE) and examined linalool odour-induced analgesia. Intraperitoneal administration of 3-methylindole (3-MI) causes a disruption of the OE and olfactory function that continues for at least three weeks^21^. Two weeks after 3-MI administration, we assessed the effects of linalool vapour exposure using the formalin test. The effects of linalool vapour exposure were not observed in 3-MI induced anosmic mice (Fig. 3C,D), indicating that the olfactory input evoked by linalool vapour was essential for inducing analgesic effects.

### Linalool odour-induced analgesia is mediated by hypothalamic orexin neurons

**Figure 4:**
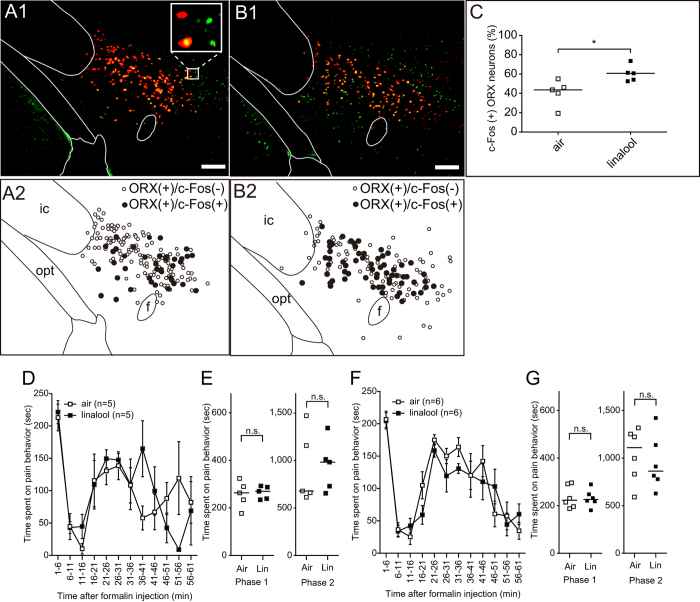
Orexinergic transmission is essential for linalool odour-induced analgesia. (**A**) Linalool odour exposure activates hypothalamic orexin neurons. Immunohistochemical analyses indicate that a subpopulation of orexin neurons (red) expressed c-Fos (green), even under odourless-air exposure (A1). The distributions of c-Fos positive (black dots) and c-Fos negative (white dots) orexin neurons are shown in (A2). (**B**) During linalool exposure, c-Fos expressing orexin neurons are widely distributed in the hypothalamus (B1, B2). (**C**) A population analysis indicates the number of c-Fos expressing orexin neurons significantly increased in the linalool-exposed group (*p* < 0.05). (**D,F**) Time course of formalin-evoked pain responses among orexin neuron-ablated (AB) mice (**D**) and orexin peptide-deficient (KO) mice (**F**). Attenuation of pain behaviours under linalool odour exposure was not observed in both mutant mouse strains. (**E,G**) Population analyses reveal linalool-induced analgesia disappearance in the orexin mutant mice. Traces are presented as mean ± SEM. Bars in plots indicate the median of each group. Air, odourless air-exposed mice; Lin, linalool-exposed mice; *n* = 5 in each group in (**A–C**); *n* = 5 for AB mice, *n* = 6 for KO mice in (**D–G**); f, fornix; ic, internal capsule. Bars in pictures indicate 200 μm.

To address the central neuronal circuits responsible for linalool odour-induced analgesia, we assessed contributions from hypothalamic orexin neurons, one of the key mediators for inducing analgesia^13^. First, we examined the activation of hypothalamic orexin neurons during linalool odour exposure through the expression of c-Fos, an immediate early gene product and marker of neuronal activation, using immunohistochemistry. After linalool odour exposure, quantitative analysis of the number of c-Fos-expressing orexin neurons indicated a significant increase in the hypothalamus (*U* = 2, *p* < 0.05, Fig. 4A–C).

Next, we examined the contribution of orexin neurons to linalool odour-induced analgesia using orexin neuron-ablated mice (AB mice) whose orexin neurons were genetically eliminated^22^. The time course of formalin-induced pain responses among linalool odour-exposed AB mice and odourless air-exposed AB mice was similar (Fig. 4D). Quantitative analyses showed that the duration of pain responses in the first and second phase were not significantly different between groups (Fig. 4E), indicating orexin neurons were essential for linalool odour-induced analgesia.

To confirm the contribution of orexinergic transmission to linalool odour-induced analgesia, we performed the formalin test on orexin peptide-deficient mice (KO mice)^23^ during linalool odour exposure. Results showed that the analgesic effects of linalool odour exposure disappeared in KO mice (Fig. 4F,G), suggesting that orexinergic transmission played a key role in linalool odour-induced analgesia.

### Linalool exposure does not induce aversive stress responses

**Figure 5:**
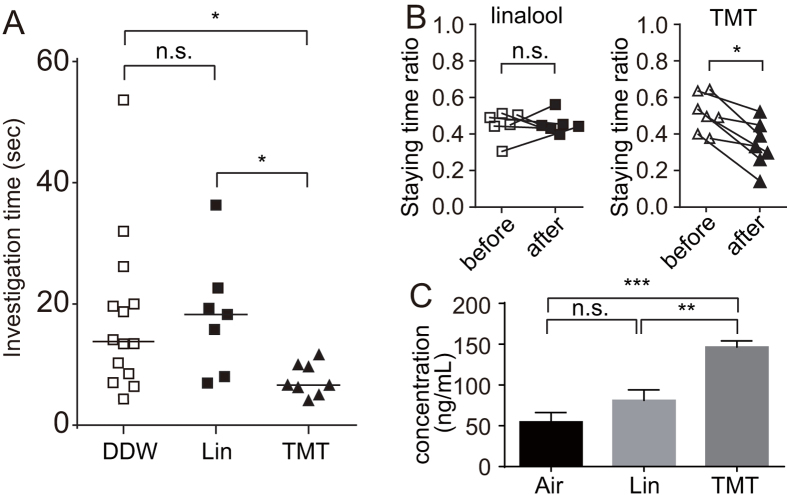
Linalool odour exposure does not induce aversive stress responses. (**A**) Innate odour preference test. Time spent investigating the odourant-scented filter paper is plotted. Kruskal-Wallis test reveal the significant difference among the three groups (*H* = 8.447, *p* < 0.05). Bars indicate the median. *n* = 14 for DDW group, *n* = 7 for linalool (Lin) group, *n* = 8 for TMT group (*n* = 8). *p < 0.05; n.s., not significant. (**B**) Innate odour avoidance test. Staying time ratio (see Methods) before and after linalool (B *left*) or TMT (B *right*) ventilation is shown. *n* = 6 (linalool), *n* = 7 (TMT). *p < 0.05; n.s., not significant (Wilcoxon matched pairs test). (**C**) Corticosterone assay. Plasma corticosterone concentration after 60 min odour exposure is shown. Data are represented as mean ± SEM. *n* = 8 for Air group, *n* = 7 for linalool (Lin) group, *n* = 8 for TMT group). **p < 0.01; ***p < 0.001; n.s., not significant (ANOVA with post-hoc Tukey’s multiple comparisons test).

To examine whether linalool odour exposure induced strong stress responses, we performed two distinct behavioural tests. An innate odour preference test showed that time spent sniffing paper scented with 2,4,5-trimethylthiazoline (TMT), a chemical from fox faeces whose odour induces stress in rodents, was significantly shorter (*p* < 0.05) than time spent sniffing paper scented with double distilled water (DDW). In contrast, this was not the case with linalool (Fig. 5A), indicating that mice did not avoid the filter paper scented with linalool but did avoid a paper with TMT. In addition, the innate odour avoidance test (Fig. 5B) showed that the staying time ratio was not significantly altered for the linalool group but was altered in the TMT group (*W* = 28, *p* < 0.05). Mice did not avoid staying in the chamber ventilated with linalool odour; linalool odour exposure does not appear to induce aversive stress in mice.

Next, to examine whether linalool odour exposure induced hormonal stress responses, we measured the plasma corticosterone (CORT), one of the stress hormones increased in the blood after stress exposure. The plasma CORT level was significantly different among the three groups (*F* (2, 20) = 20.31, *p* < 0.0001); it was slightly but not significantly increased in the linalool-exposed group, whereas the increase was significant in the TMT-exposed group (p < 0.0001) (Fig. 5C). Thus, linalool odour exposure does not appear to induce strong stress responses in mice.

## Discussion

### Olfactory input evoked by linalool odour triggers analgesia

In the present study, we performed the hot plate test under odour exposure as an initial screening of potentially analgesic odour molecules. In our experimental condition, only linalool odour showed significant extension of the response latency compared with the odourless air control. Furthermore, the latency of the linalool group tended to be extended compared with the other five odourants (Fig. 1A). Therefore we focused on and further examined the effect of linalool.

To examine whether the olfactory input mediates the linalool odour-induced analgesia, we assessed the analgesic effects in olfactory bulbectomized mice. In the bulbectomized mice, the basal pain behaviours were seemed to be reduced (Fig. 3A and B). We could not explain the exact reason of the observation. Because the basal stress level of the bulbectomized mice is still under debate in terms of the circular corticosterone level^24^,^25^, it is not likely that the observation resulted from stress-induced analgesia caused by bulbectomy. However, recent behavioural studies have raised the possibility that olfactory bulbectomy may cause depression-like behaviours in rodents^24^,^25^, possibly affecting pain behaviours. Therefore we confirmed the effect of olfactory deprivation in mice with 3-MI-induced OE disruption. To our knowledge, there are no reports showing that treatment with 3-MI induces depression-like behaviours in mice.

In contrast to previous studies, we showed for the first time that the olfactory input evoked by the linalool odour had an analgesic effect (Fig. 3), and that the central olfactory system and hypothalamic circuits mediated this effect. Several previous studies have shown that subcutaneous^1^,^2^,^3^,^26^, intraperitoneal^4^,^5^, intrathecal injection^5^, or oral administration^5^ of linalool attenuated pain responses. While the underlying mechanisms have not been fully elucidated, linalool might directly affect glutamatergic transmission via ionotropic glutamate receptors in the central nervous system^5^,^26^. Our findings support a potential therapeutic approach for treating pain with linalool odour stimulation.

### Linalool-induced analgesia is not aversive stress-induced analgesia

It has long been known that predator odours^27^,^28^,^29^ and several odour molecules derived from predators^30^,^31^ induce analgesic effects in rodents. TMT, for example, induces strong analgesic effects^30^,^32^. Because predator odour stimulation accompanies various stress-related responses, including freezing and avoidance behaviour and increases in plasma stress hormones, predator odour-induced analgesia is considered a form of stress-induced analgesia^33^. Analogous to predator odour-induced analgesia, linalool odour-induced analgesia could also be considered a form of stress-induced analgesia; we propose that it is not. Odour preference/avoidance tests showed that mice did not show aversive responses to linalool but did to TMT (Fig. 5A,B). In addition, plasma corticosterone levels did not show a significant increase after linalool odour exposure but did after exposure to TMT (Fig. 5C). Thus, linalool odour stimulation likely induced analgesic effects without fearful stress.

### Possible central olfactory pathway triggering hypothalamic orexin neurons for linalool-induced analgesia

The present study revealed that orexin neurons were essential for linalool odour induced analgesia (Fig. 4). However, the neuronal pathway activating the orexin neurons and contributing to analgesia remains unknown. Previous tracing studies revealed that hypothalamic orexin neurons received direct synaptic input from various brain regions, including the amygdaloid nuclei (anterior cortical amygdaloid nucleus, basolateral amygdaloid nucleus, basomedial amygdaloid nucleus, bed nucleus of the stria terminalis, central nucleus, and medial amygdaloid nucleus), septal area (lateral septal nucleus and medial septal nucleus), and medial prefrontal cortex (prelimbic cortex and infralimbic cortex)^34^,^35^. Among these brain regions, the anterior cortical amygdaloid nucleus receives massive axonal input from the main olfactory bulb and olfactory cortices^36^,^37^. Furthermore, other deep nuclei in the amygdala receive synaptic inputs from the olfactory cortices^38^. Therefore, the amygdaloid nuclei are candidates for relaying the olfactory input that drives linalool-induced analgesia.

### Possible analgesic pathway driven by linalool-activated orexin neurons in the hypothalamus

The output of the orexin neurons activated by linalool odour stimulation has yet to be confirmed. Three analgesic pathways are plausible: direct suppression of nociceptive information by orexinergic transmission in the spinal cord, indirect suppression through activation of the descending inhibitory pathway, and anti-nociception induced by activation of the ventral tegmental area (VTA). Hypothalamic orexin neurons have direct axonal projections to lamina I of the spinal cord^39^,^40^ and spinal cord neurons express orexin-1 receptors^14^,^41^. Consistent with anatomical observations, intra-spinal administration of orexin peptide attenuates pain responses in rodents^14^,^42^,^43^,^44^. Thus, linalool-activated orexin neurons might directly suppress nociceptive neurons in the spinal cord. Conversely, orexin neurons have massive axonal projections to the periaqueductal gray matter (PAG)^45^,^46^, which controls the serotonergic descending pain inhibitory pathway^47^. Because direct administration of an orexin receptor agonist into the PAG induces significant analgesic effects^48^,^49^, orexinergic activation of the PAG-descending pain inhibitory pathway might be involved in linalool-induced analgesia. Orexin neurons can also induce anti-nociception by modulating the VTA-nucleus accumbens (NAc) pathway. Intra-VTA administration of orexin-A induces anti-nociception in a dose-dependent manner^50^. Moreover, intra-VTA administration of an orexin-1 receptor antagonist prevents lateral hypothalamus stimulation-induced anti-nociception^51^. Because orexinergic modulation of the VTA-NAc pathway plays a pivotal role in motivated behaviours^52^,^53^, linalool odour stimulation may trigger the analgesic pathway incorporated in a battery of physiological processes related to those motivated behaviours.

In conclusion, we observed linalool odour-induced analgesia mediated by hypothalamic orexin neurons. This is, to our knowledge, the first report showing that olfactory input induces an analgesic effect without aversive stress in rodents. Though the biological role of linalool-odour induced analgesia is not yet fully understood, our findings support a potential therapeutic approach for treating pain with linalool odour stimulation.

## Methods

### Animals

Wild type mice (C57BL/6, 24–34 g, *n* = 181), orexin neuron-ablated (AB) mice (25–34 g, *n* = 10), and orexin peptide-deficient (KO) mice (34–43 g, *n* = 12) were used. The generation of mutant mice has been described in detail elsewhere^22^,^23^. Mutant mice were maintained as heterozygotes and crossed to obtain null mutants. We backcrossed the mutant mice with C57BL/6 mice (Clea Japan Inc., Tokyo, Japan) for more than 10 generations. All experiments were performed in male mice to avoid possible differences related to menstrual cycling in females. Animals were housed with lights on at 7:00 A.M. and off at 7:00 P.M. All experiments were performed during the light cycle, between 1:00 P.M. and 6:00 P.M. All experiments were performed in accordance with guidelines outlined by the Physiological Society of Japan and were approved by the Experimental Animal Research Committee of Kagoshima University.

### Odour exposure

Odour exposure was performed with a custom-made olfactometer. Briefly, 0.5 mL of an odourant was dispensed into an uncapped glass vial (diameter: 27.5 mm, content: 20 mL). The vial was placed into the odour chamber (0.32 L), and the odourant molecule was vaporized at room temperature (25 ±1 °C). Clean air deodorized with a charcoal filter and double distilled water (DDW) were introduced to the odour chamber from a compressed air cylinder, and output odorous air was used for ventilation in the observation chamber (top diameter: 8 cm, base diameter: 11.5 cm, height: 15 cm, content: 1 L) at a constant rate (1 L/min). After 5 min pre-ventilation of odorous air, a mouse was individually placed into the observation chamber ventilated with odorous air. Because the humidity of the carrier gas and the temperature of the odour chamber were kept constant, the concentration of the odorous gas was considered constant.

### Odourants

Odourants were purchased from Tokyo Chemical Industry (linalool, 1,8-cineol, linalyl acetate, myrcene, β-pinene, eugenol) or from Phoenix Pharmaceuticals (2,4,5-trimethylthiazoline, TMT). All odourants were stored at 4 °C and dispensed into a glass cup during each trial to prevent odourant degradation. Odourless mineral oil used for dilution of odourants was purchased from Sigma-Aldrich.

### Olfactory bulbectomy

For deprivation at the olfactory bulb level, we performed a bilateral olfactory bulbectomy (OB)^54^. Mice were deeply anesthetized with an intraperitoneal injection of a ketamine and xylazine (100 mg/kg and 10 mg/kg, respectively) cocktail. Bilateral olfactory bulbs were aspirated with a blunt intravenous catheter attached to a 5-mL syringe. To prevent infection, mice were given 40,000 U/kg of penicillin G by subcutaneous injection. The OB mice were allowed to recover for two weeks after surgery. Complete removal of bilateral olfactory bulbs without damage to the frontal cortex was confirmed with a fixed brain sample after a behavioural test.

### Olfactory epithelium deprivation

For olfactory epithelium deprivation, we treated mice with 3-MI, which induces extensive destruction of the olfactory mucosa, resulting in anosmia^21^. Briefly, 300 mg/kg of 3% 3-MI in corn oil was administered by intraperitoneal injection. Two weeks after the injection, 3-MI mice were used for the behavioural test.

### Pain Assays

To assess the thermal pain threshold, we performed a classical hot plate test using electronically controlled hot plate apparatus (#7280; Ugo Basile) heated to 54.5 °C. A mouse was placed in the observation chamber and exposed to odour vapour for 5 min. After odour exposure, the mouse was placed on the hot plate and covered with the observation chamber ventilated with odorant vapour. The latency before the animal licked, shook, or fluttered its hind paw, or jumped on the hot plate was recorded. To prevent the mouse from being injured, a cutoff time was set to 1 min.

To assess pain behaviour induced by irritant, we performed a formalin test. First, mice were exposed to odourant vapour for 5 min in the observation chamber. After the odour exposure, 20 μL of 2% formalin was subcutaneously injected into the plantar surface of the left hind paw within 1 min. The mouse was then put back into the observation chamber ventilated with odourant vapour, and time spent performing pain behaviours (licking, biting, flinching, and lifting of the injected paw) was measured. Beginning 1 min after the formalin injection, cumulative duration of pain behaviour was measured every 5 min up to 1 h.

To confirm analgesic effects of morphine injection for OB mice, 10 mg/kg of morphine was provided subcutaneously in the neck 30 min before the formalin injection.

### Spontaneous activity test

To assess whether the linalool vapour exposure induce motor impairment or not, we examined the spontaneous activity under the odour exposure. A digital video camera was mounted at the ceiling of the observation chamber and the spontaneous activity of mice under the odour exposure was recorded during 6 min (corresponding to the sum of 5 min pre-exposure and 1 min hot plate test). Then the video data was analysed by EthoVision XT software (Noldus Information Technology, Wageningen, Netherlands) to measure the total distance moved. In addition, the number of rearing was counted manually from the video data.

### Immunohistochemistry

To assess activation of hypothalamic orexin neurons induced by linalool odour stimulation, orexin and c-Fos were double-labelled using fluorescence immunohistochemistry^15^. Mice were exposed to linalool odour using the olfactometer for one hour. Immediately after odour exposure, mice were deeply anesthetized with urethane (1.3 g/kg, i.p.), and perfused transcardially with saline followed by 4% paraformaldehyde in 0.01 M PBS (pH 7.4). The brain was then removed and post fixed at 4 °C overnight. Coronal sections, including the hypothalamus, were cut at 40-μm thickness using a vibratome and collected in PBS, and we performed floating immunohistochemical staining^51^. Sections were sequentially incubated with PBS containing 0.3% Triton-X and 1% normal donkey serum for 30 min, rabbit anti c-Fos antiserum (1/1000, Oncogene Research Products, San Diego, CA, USA) for 60 min, biotinylated anti-rabbit IgG antibody (1/250, Jackson ImmunoResearch, West Grove, PA, USA) for 90 min at room temperature, and streptavidin conjugated Alexa Fluor 488 (1/200, Invitrogen, Eugene, OR, USA) for 90 min in a dark box. Subsequently, sections were incubated with goat anti orexin-A antiserum (1/100, Santa Cruz Biotechnology, Santa Cruz, CA, USA) and CF568-conjugated anti goat IgG (1/200, Biotium, Heyward, CA, USA) for 90 min in a dark box. These sections were mounted on a slide glass and examined under a fluorescent microscope. The number of c-Fos positive cells was counted for three stained sections through the hypothalamus in a blinded manner to the treatment (linalool or air).

### Odour preference test

An odour preference test was adopted from a previously published procedure^55^. For acclimatization, mice were placed in a test cage (20 × 15 × 13 cm) for 30 min and transferred to a new cage four times for each animal. After the acclimatization process, mice were transferred to a new cage, and a filter paper (2 × 2 cm) scented with a test odourant was introduced. Time spent investigating the filter paper during a 3-min test period was measured. Odourants used were linalool (100 μM), TMT (857 μM), and saline.

### Odour avoidance test

For the odour avoidance test, we used a custom made apparatus composed of three chambers connected with mesh tubes, which minimized the mixing of odourant vapours from each chamber, as described in detail elsewhere^56^. The side chambers were ventilated (1 L/min) with either odourant vapour produced in the olfactometer or odourless air.

First, mice freely explored the apparatus for 60 min during acclimatization. On the second day, we tested whether mice preferred a specific side of the chamber while both chambers were ventilated by odourless air. No animal had an initial bias for either chamber. A few days after the initial session, an avoidance test was performed. One side chamber was randomly selected to be ventilated with odourant vapour and another side with odourless air. The subsequent behaviour was observed for 10 min.

Time spent in each side of the chamber was recorded to assess aversion to the odour-ventilated chamber. The time ratio was the amount of time spent in the odour-ventilated chamber divided by the total amount of time spent in both chambers. Therefore, the ratio would be 0.5 in a case where a mouse spent the same amount of time in both chambers, and the ratio would be 0.2 in a case where the time spent in the odour-ventilated chamber was four fold less than the odourless chamber. The time ratio during a check-up trial was adopted for a pre-exposure control. The ratio difference between control and odourant exposure trials was assessed with a Wilcoxon test.

### Corticosterone ELISA

Mice were housed individually to avoid increases in plasma corticosterone due to conflict. After odour exposure with the custom made olfactometer for 60 min, mice were decapitated quickly, within 15 sec of removing them from the olfactometer, and blood samples were collected in an EDTA tube (microtainer 365974, BD, NJ, USA). The plasma was promptly separated in a refrigerated centrifuge (4 °C) and stored at −20 °C. Plasma corticosterone concentrations were measured using a corticosterone ELISA kit (ADI-900–097, ENZO, NY, USA) according to the manufacture’s protocols.

### Data analyses

If not otherwise specified, statistical comparisons were performed using the Kruskal-Wallis test with post-hoc Dunn’s multiple comparison test (for comparisons of more than three groups) or the Mann-Whitney U test (between two groups) using Prism6 software (GraphPad Software, Inc.). The criterion for statistical significance was *p* < 0.05 in all cases. Traces of pain behaviour during the formalin test are presented as mean ± SEM. Bars in the plots indicate the median.

## Additional Information

**How to cite this article**: Tashiro, S. *et al*. Odour-induced analgesia mediated by hypothalamic orexin neurons in mice. *Sci. Rep.* **6**, 37129; doi: 10.1038/srep37129 (2016).

**Publisher’s note:** Springer Nature remains neutral with regard to jurisdictional claims in published maps and institutional affiliations.
